# Evaluation of modified window-based scatter compensation in quantitative ^177^Lu-SPECT for a ring-configured CZT SPECT-CT

**DOI:** 10.1186/s40658-026-00905-0

**Published:** 2026-06-14

**Authors:** Irma Ceric Andelius, Anna Stenvall, Elias Nilsson, Erik Larsson, Johan Gustafsson

**Affiliations:** 1 Radiation Physics, Department of Haematology, Oncology, and Radiation Physics, Skåne University Hospital, Lund, Sweden; 2 Department of Translational Medicine and Wallenberg Centre of Molecular Medicine, Lund University, Malmö, Sweden; 3 Medical Radiation Physics, Lund University, Lund, Sweden

**Keywords:** Quantitative SPECT, CZT, Ring-configured SPECT, Lutetium-177, Scatter compensation

## Abstract

**Background:**

The aim was to evaluate bias and precision for ^177^Lu-activity-quantification using window-based scatter compensation in a ring-configured CZT gamma camera (StarGuide™, GE HealthCare). This paper extends a previous study by applying modified scatter compensation (MSC) methods introduced with the StarGuidePlus upgrade. The modified methods account for contamination of scatter windows by primary photons due to tailing. Whilst the original compensation addressed tailing in the 208 keV peak only, the upgrade extends the concept to the 113 keV peak and enables the estimated primary signal in the scatter windows to be included to the measured projections (MSC + P).

**Methods:**

Listmode-data for a NEMA phantom and anthropomorphic phantom were reframed to accommodate the updated settings for MSC and MSC + P. Reconstruction was performed using OS-EM (two to 30 iterations, 10 subsets) with compensation for attenuation, scatter (DEW/TEW, MSC and MSC + P), spatial resolution, and penetration at 208 keV. Volumes-of-interest following the manufacturer-specified sphere sizes were defined and activity-concentration was quantified for each sphere and the total phantom activity across six (10 min) time-frames. Bias was evaluated with mean relative error and precision with coefficient of variation (CV).

**Results:**

Imaging at 208 keV generally results in similar activity concentrations and total activity estimates for MSC and MSC + P (mean relative error: − 24.8 to − 23.4%, CV: 0.6 and 0.7% for the larges sphere). For 113 keV, a slightly better precision is obtained for MSC and MSC + P (mean relative error: − 15.4 and − 21.5%, CV: 0.6 and 0.7% for the largest sphere in the NEMA phantom) but a larger bias compared with TEW (mean relative error − 7.1%, CV 0.8%). The major difference is for total activity where the MSC and MSC + P decrease bias compared to TEW (5 and 4% versus 21% for 113 keV in the NEMA phantom).

**Conclusions:**

The improvement in activity-concentration accuracy in regard to bias and precision is modest in small-volume high activity-concentration regions. However, the findings suggest potential value in preservation of total activity for both MSC and MSC + P.

## Background

Quantitative single photon emission computed tomography (SPECT) is the cornerstone of image-based dosimetry in radionuclide therapy [1], with low bias and good precision of estimated activity concentrations being necessary for reliable and reproducible dosimetry. The introduction of clinical semi-conductor cadmium-zinc-telluride (CZT) ring-configured gamma-camera has led to an interest in using these systems also for quantitative tasks both for diagnostic (e.g., ^99m^Tc) and therapeutic (e.g., ^177^Lu) radionuclides [2].

The radionuclide ^177^Lu emits two gamma photons useful for SPECT imaging at 113 keV and 208 keV. However, imaging of this radionuclide using CZT systems poses challenges as the systems are primarily designed for diagnostic tasks using ^99m^Tc, and the need for alignment between collimator holes and anode elements limits the practicality of frequent collimator exchange. Thus, imaging at the higher energy suffers from penetration [2, 3], whilst imaging at the lower energy suffers from a poor scatter-to-primary ratio. Although the poor scatter-to-primary ratio is a general issue for quantitative SPECT at this energy, its mitigation is further complicated by the low-energy tail typical for pixelated CZT detectors [4]. The charge-sharing phenomenon results in a tail of low-energy primary photons in the acquired signal, which makes window-based scatter compensation, such as the dual-energy window (DEW) and triple-energy windows (TEW) techniques, problematic. An underlying assumption of the standard DEW and TEW methods [5, 6] is that the signal in the scatter windows consists of scattered photons only, and thus any contamination of primaries from the low-energy tail will result in erroneous scatter estimates and thus biased activity concentrations in the reconstructed image [7, 8]. Hence, the use of window-based scatter compensation for quantitative SPECT with CZT cameras requires consideration of such contamination.

We demonstrated in a previous study that a ring configured CZT-system (StarGuide^TM^, GE HealthCare, Haifa, Israel), is a viable alternative to the dual headed Anger system for quantitative ^177^Lu-SPECT [2]. We further demonstrated that the ring-configured CZT system has a potential quantitative advantage compared with conventional Anger systems for imaging at 113 keV. However, we also noted substantial overestimation of total activity for imaging at this energy, which we suspected was related to imperfect scatter compensation. The present short communication extends our earlier investigation by evaluating a modified scatter-compensation (MSC) method that accounts for low energy tail of primary photons [9], enabled with the StarGuidePlus upgrade version 1.0 on the StarGuide system. Previous versions of the system implemented this method only for DEW scatter compensation at 208 keV and the upgrade for the 208 keV peak only entails different energy-window settings and k-factors used to estimate scatter and primary photons. The upgraded software also implements a TEW version applicable for imaging at 113 keV, which should reduce bias for this energy. The updated scatter-compensation routines also allow for primary photons in scatter windows to be included in the main window for the reconstruction (MSC + P), thereby boosting the signal-to-noise ratio. We aim to assess the quantitative impact at 113 and 208 keV with respect to bias and precision for MSC, and MSC + P compared with the old scatter-compensation methods.

## Materials and methods

The methodology applied here follows the approach described by Stenvall et al. [2], summarized here in brief. For a more comprehensive description, the reader is referred to the original publication. The principal aim of these projects is to assess the quantitative performance regarding the estimation of activity concentration in repeated measurements by reporting the mean relative error and the coefficient-of-variation (CV). We used the mean relative error as measure of the systematic error, and the CV as measure of the variability over repeated measurements. The data were acquired by performing one long acquisition of 60 min which is then reframe to generate six 10-minute measurements. This short communication includes an additional liver background volume of interest (VOI), extending the analysis presented in Stenvall et al. [2], were we report the mean relative error and standard deviation.

### Phantoms

Phantoms described in Stenvall et al. [2] were utilized: A uniform cylindrical phantom (SUV phantom), an image quality NEMA phantom (NEMA IEC Body phantom) and an anthropomorphic phantom (LK-S Kyoto Liver/Kidney Phantom, Kyoto Kagaku Co., Ldt.). A new set of calibration factors was determined using the uniform cylindrical phantom for MSC and MSC + P. The NEMA phantom with six spheres was used to evaluate the quantitative performance of MSC and MSC + P for ^177^Lu. The smallest sphere was replaced by a sphere with a volume of 113 ml and the spheres were positioned alternating between largest and smallest. The anthropomorphic phantom contained a liver insert with two spheres (Table 1). The sphere activity concentration and the tumour-to-liver ratio (set to 14) were based on the relationship determined from the pharmacokinetic model developed by Brolin et al. [10] 24 h post administration of [^177^Lu]Lu-DOTA-TATE. Specifications for the phantoms are provided in Table 1.

**Table 1:** Specification of the phantoms prepared further described in by Stenvall et al. [2]

	Uniform phantom	NEMA phantom	Anthropomorphic phantom
Diameter (mm)/Volume (mL) (fillable containers inner dimensions)	− /5640	Sphere N1: 13/1.2Sphere N2: 17/2.6 Sphere N3: 22/5.6 Sphere N4: 29/11.5 Sphere N5: 37/26.5 Sphere N6: 60/113.1	Sphere A1: 27/9.9Sphere A2: 36/25.3
^177^Lu activity concentration (MBq/ml)	0.098	1.8	Spheres: 1.93 (cold liver) 1.87 (warm liver) Liver: 0.13

### SPECT reconstruction and calibration

Previously collected listmode-data were reframed to adjust the energy window settings recommended for MSC and MSC + P (Table 2), and images were then reconstructed for the 113 keV and 208 keV peak separately [2]. Reconstructions were performed with the ordered subset expectation maximization (OS-EM) algorithm with 2, 5, 10, 20 and 30 iterations (10 subsets) with a voxel size of 2.46 × 2.46 × 2.46 mm^3^ using compensation for attenuation, distant-dependent resolution, scatter (MSC and MSC + P, as specified in Table 2), and compensation for penetration for the 208 keV peak. All reconstructions were performed on the Smart Console version 1.0 (GE Healthcare, Haifa, Israel). Reconstructed images from Stenvall et al. [2] were used for comparison. These were reconstructed with the same settings with respect to attenuation and resolution recovery but differed in energy window widths and scatter compensation method. The images for 113 keV was reconstructed with TEW scatter compensation (with no account for the primary tail) and the images for 208 keV was reconstructed with DEW scatter compensation but accounting for the primary tail (a setting that could not be disabled). These two methods will be referred to the names used in Stenvall et al. [2], i.e., TEW and DEW.

The calibration factor was determined by placing a large cylindrical VOI (radius 7 cm and depth 20 cm) in the centre of the reconstructed image. For a thorough description, the reader is referred to Stenvall et al. [2].

### Scatter compensation

Scatter compensation was employed by acquiring multiple energy windows (primary window (PW), low window (LW), high window (HW)). The MSC assumes that all energy windows contain primary and scattered photons. The problem is formulated as a linear system of equations using predetermined weighting factors obtained through calibration. By solving this equation system, pure scatter and primary contributions can be estimated for each window. The scatter estimates are added to the forward projection in the iterative reconstruction [9]. MSC + P incorporates counts outside the primary window, including contribution from primaries in the scatter window, boosting the signal-to-noise ratio [9]. Details about the energy window settings are presented in Table 2.

**Table 2:** Energy window settings

Type	Window centre/keV	Window width/%
DEW and TEW		
Emission (113 PW)	113.0	± 10
Scatter (113 LW)	96.6	− 4.6/+4.4
Scatter (113 HW)	129.4	− 3.3/+3.7
Emission (208 PW)	208	± 6
Scatter (208 LW)	185	± 5
MSC and MSC + P		
Emission (113 PW)	113.0	± 8
Scatter (113 LW)	93.5	± 11
Scatter (113 HW)	133	± 8
Emission (208 PW)	208	± 6
Scatter (208 LW)	182.7	± 7

PW: primary window, LW: low window, HW: high window

### Evaluation

Spherical VOIs were defined by identifying the centre-of-mass for each of the six spheres in the SPECT images of the NEMA-phantom, and then including the voxels closest to the centre-point until the physical sphere volume was reached. The same VOIs were used for all time-frames. For the anthropomorphic phantom with warm liver background, a 10 ml spherical VOI was also defined in the liver background, located to avoid influence for partial-volume effects. The same set of VOIs were used for all reconstructions. The bias was characterised by the mean relative error of the activity concentration over timeframes

1 $$\:{\epsilon\:}_{i}=\frac{{\stackrel{-}{C}}_{i}}{{C}_{\mathrm{r}\mathrm{e}\mathrm{f}}}-1,$$

where $\:{\stackrel{-}{C}}_{i}$ is the average of the measured activity concentration for sphere *i*, and $\:{C}_\mathrm{ref}$ is the calculated activity concentration at the start of image acquisition. The CV was computed, as a measure of precision, according to

2 $$\:CV=\frac{{s}_{i}}{{\stackrel{-}{C}}_{i}},$$

where $\:{s}_{i}$ is the standard deviation in the calculated activity concentration for sphere *i* over the six timeframes.

The estimation of total activity in each phantom was studied by defining a VOI for the whole phantom body. The activity estimated from images was compared with the activity determined from phantom preparation.

## Results

The results presented for MSC and MSC + P are compared with DEW and TEW, originally reported in Stenvall et al. [2] and included here to enable direct comparison. This is true for all comparisons except the liver VOI, extending the analysis presented in Stenvall et al. [2]. Unless otherwise stated “all methods” refer to methods DEW/TEW, MSC and MSC + P.

Image calibration factors generated from the cylindrical uniform phantom over iterations were [min, max]: [80.7 s^−1^ MBq^−1^, 91.6 s^−1^ MBq^−1^] (TEW, 113 keV), [140.4–141.3 s^−1^ MBq^−1^] (MSC, 113 keV), [262.3–265.8 s^−1^ MBq^−1^] (MSC + P, 113 keV), [98.9–105.2 s^−1^ MBq^−1^] (DEW, 208 keV), [96.9–101.1 s^−1^ MBq^−1^] (MSC, 208 keV) and [128.4–135.9 s^−1^ MBq^−1^] (MSC + P, 208 keV).

### NEMA phantom

**Fig. 1:**
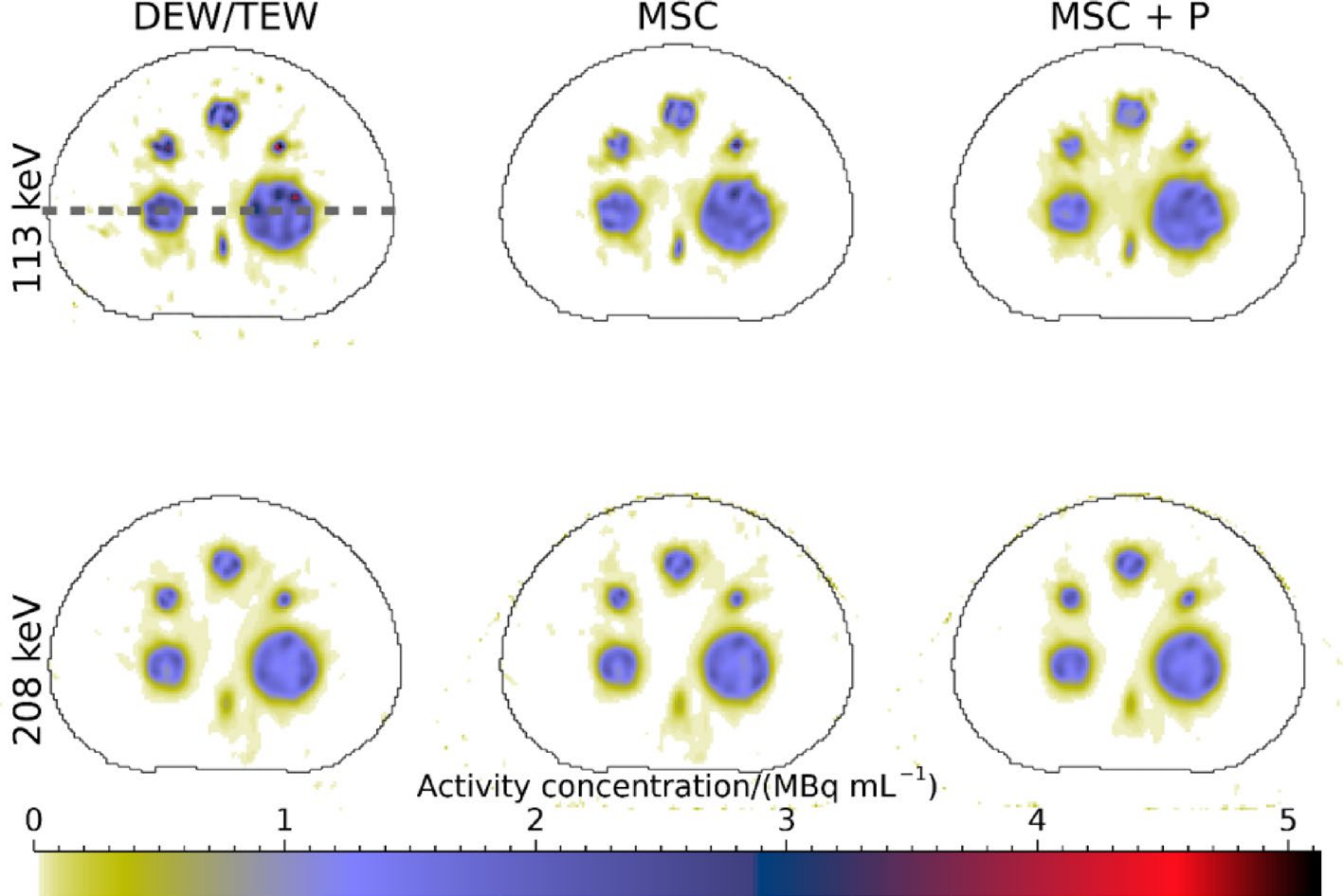
Transversal slice of the NEMA phantom reconstructed with 10 iterations (10 subsets). The dashed line in the image for DEW/TEW at 113 keV indicates the location of the profile in Fig. 2. Images for DEW and TEW are for data from Stenvall et al. [2] and are included for comparison

**Fig. 2:**
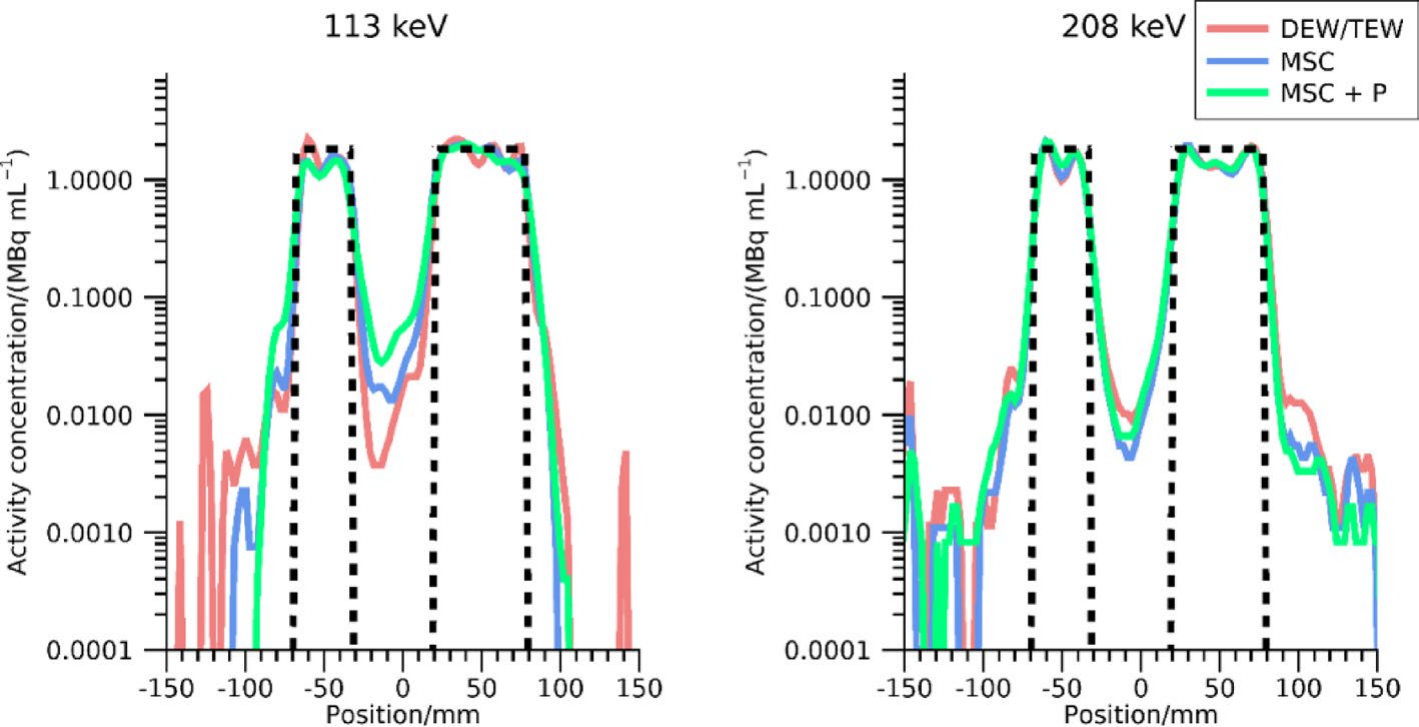
Profiles trough sphere 5 (26.5 ml) and sphere 6 (113 ml) plotted on a logarithmic scale reconstructed with 10 iterations. The dashed line indicates the true activity concentration. Data for DEW and TEW are from Stenvall et al. [2] and are included for comparison

Examples of reconstructed images for 113 and 208 keV are presented in Fig. 1. The 208 keV images reconstructed with DEW, MSC and MSC + P appear visually similar, whilst the 113 keV images differ depending on scatter compensation method. The TEW and MSC images have a better contrast than the MSC + P image where a low-signal background is introduced between the spheres. The dashed line in Fig. 1 indicates a profile through the two largest spheres and the non-radioactive background reconstructed with 10 iterations and 10 subsets that are presented in Fig. 2. The profiles show signal outside of the spheres for all scatter compensation methods and for both energies. For the 208 keV peak with DEW a slightly increased signal outside the spheres was observed whereas for MSC and MSC + P there is no apparent difference. For the 113 keV peak, a difference is seen when comparing TEW with MSC and MSC + P.

**Fig. 3:**
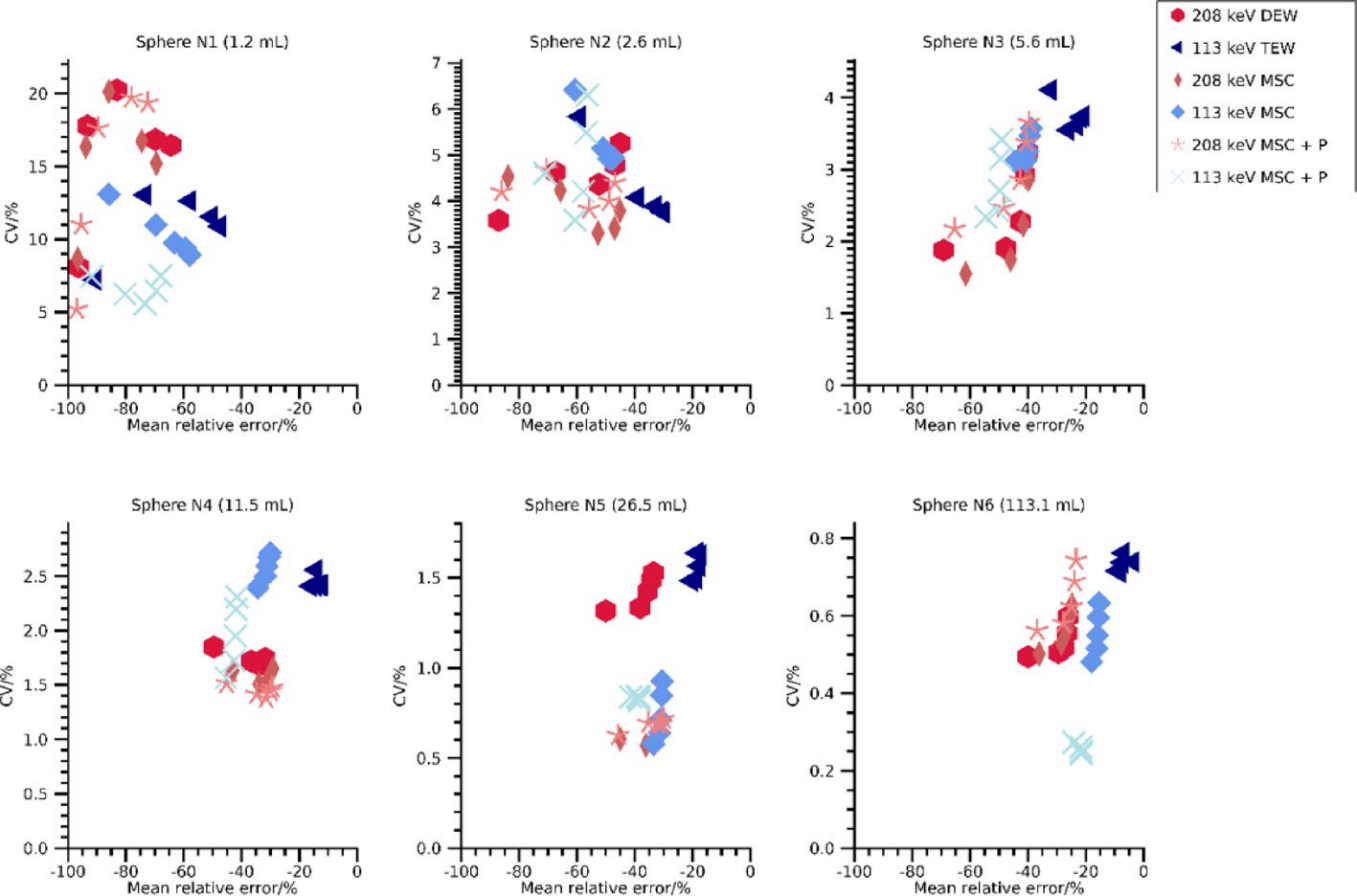
Coefficient of variation versus mean relative error for NEMA phantom where each symbol represents a specific number of iterations (2, 5, 10, 20, 30 (10 subsets)). Note that the ordinate is individual for each subplot. Data for DEW and TEW are from Stenvall et al. [2] and are included for comparison

Plots of mean relative error versus CV for different reconstructions are shown in Fig. 3 and numerical data at 30 iterations are presented in Table 3. There is a tendency of lower CV for increasing sphere size (e.g., 15% for sphere N1 to 0.6% for sphere N6 for MSC 30 iterations at 208 keV). For the 208 keV peak for DEW, MSC and MSC + P the mean relative errors and CV result in similar values (Table 3). For the 113 keV peak, the mean relative error is higher, but the CV is clearly reduced when using MSC and MSC + P compared to TEW. This reduction is most pronounced for MSC + P, which yields a 0.6% decrease in CV compared to TEW (e.g., sphere N6 mean relative error − 15.4 and − 21.5% versus − 7.1 and CV 0.6 and 0.2% versus 0.8% at 30 iterations).

**Fig. 4:**
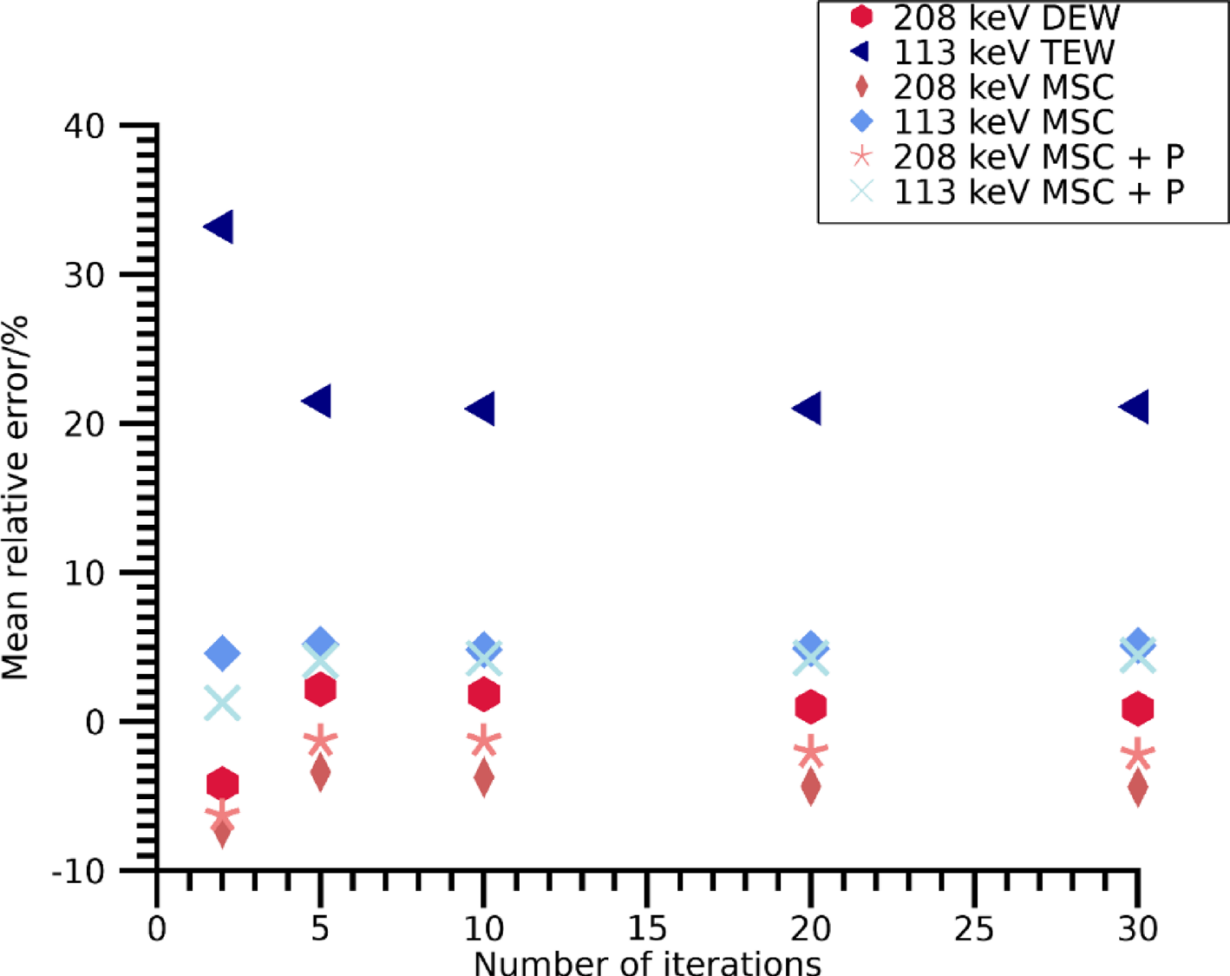
Mean relative error in estimated total activity in the NEMA phantom for the 113 keV peak for TEW, MSC and MSC + P and for the 208 keV peak for DEW, MSC and MSC + P as a function of number of iterations (2, 5, 10, 20, 30 (10 subsets)). Data for DEW and TEW are from Stenvall et al. [2] and are included for comparison

Mean relative errors in estimated total activity as a function of number of iterations are shown in Fig. 4. For both the 113 keV and the 208 keV images, the total activity is stabilized after approximately 5 iterations (i.e., 50 updates) for all scatter compensation methods (TEW/DEW, MSC and MSC + P). For the 208 keV peak, a bias of about 4% (DEW) and − 4% (MSC) are obtained whereas MSC + P has a mean relative error of about zero for estimation of total activity within the evaluated dataset. When using TEW for the 113 keV peak, the results demonstrate a systematic overestimation of about 20% (TEW) whereas the remaining methods result in mean errors within 10% (MSC and MSC + P).

**Table 3:** Mean relative error (CV) for estimated activity concentration in the NEMA and anthropomorphic phantom at 30 iterations

Sphere		113 keV			208 keV		
	Volume/ml	TEW/%	MSC/%	MSC + *P*/%	DEW/%	MSC/%	MSC + *P*/%
N1	1.2	− 48 (11)	− 57.9 (8.9)	− 67.9 (7.5)	− 64 (16)	− 69 (15)	− 72 (19)
N2	2.6	− 29.9 (3.7)	− 47.5 (5.0)	− 56.4 (6.3)	− 45.1 (5.3)	− 45.1 (3.8)	− 46.9 (4.4)
N3	5.6	− 20.9 (3.7)	− 38.9 (3.6)	− 49.1 (3.4)	− 40.1 (3.2)	− 38.9 (3.2)	− 39.7 (3.7)
N4	11.5	− 12.4 (2.4)	− 30.1 (2.7)	− 41.6 (2.3)	− 31.8 (1.7)	− 29.1 (1.7)	− 29.3 (1.5)
N5	26.5	− 17.4 (1.6)	− 30.5 (0.9)	− 38.4 (0.8)	− 33.5 (1.5)	− 31.3 (0.7)	− 30.0 (0.7)
N6	113.1	− 7.1 (0.8)	− 15.4 (0.6)	− 21.5 (0.2)	− 26.1 (0.6)	− 24.8 (0.6)	− 23.4 (0.7)
A1 C	9.9	− 20.5 (1.8)	− 37.5 (2.0)	− 48.3 (1.5)	− 38.3 (3.2)	− 36.8 (3.1)	− 36.5 (1.8)
A1 W	9.9	− 23.1 (5.7)	− 35.5 (5.6)	− 43.8 (4.0)	− 39.7 (1.6)	− 39.2 (1.7)	− 39.5 (1.0)
A2 C	25.3	− 11.7 (1.9)	− 28.2 (2.1)	− 39.2 (1.6)	− 31.6 (0.5)	− 29.3 (0.7)	− 29.2 (0.7)
A2 W	25.3	− 16.3 (2.6)	− 27.1 (1.1)	− 34.3 (1.5)	− 32.5 (2.2)	− 30.7 (2.3)	− 29.4 (1.6)

The NEMA spheres are denoted N1-N6 and the spheres in the anthropomorphic phantom are denoted A1-A2 (C for cold background and W for warm background). Data for DEW and TEW are from Stenvall et al. [2] and are included for comparison.

### Anthropomorphic phantom

**Fig. 5:**
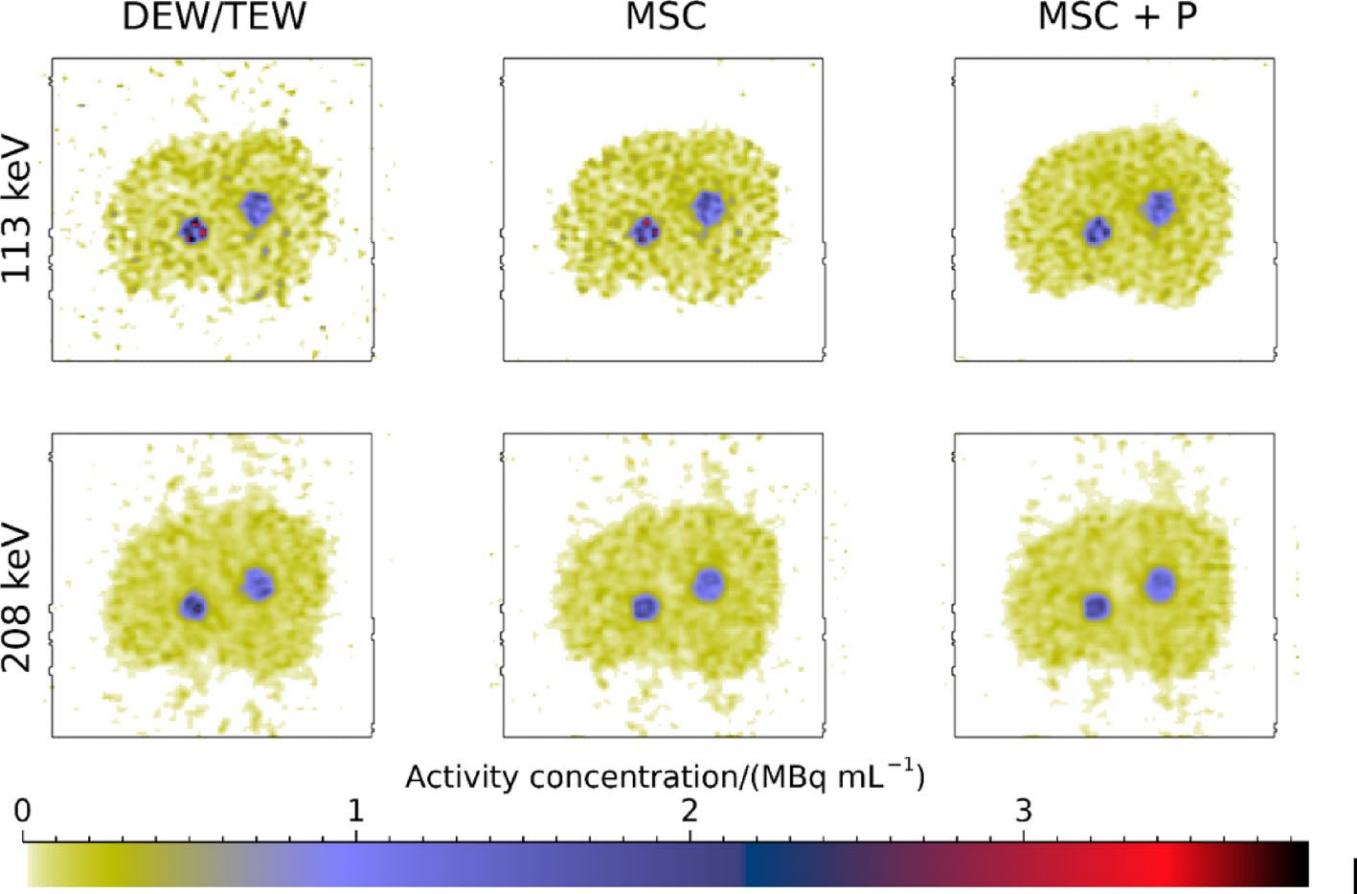
Sagittal slice for the anthropomorphic phantom with warm background reconstructed with 10 iterations (10 subsets). Top row shows images reconstructed with 113 keV (top left: TEW peak, middle: MSC, top right: MSC + P) and bottom row shows images reconstructed with 208 keV (top left: DEW, middle: MSC, top right: MSC + P). Images for DEW and TEW are for data from Stenvall et al. [2] and are included for comparison

**Fig. 6:**
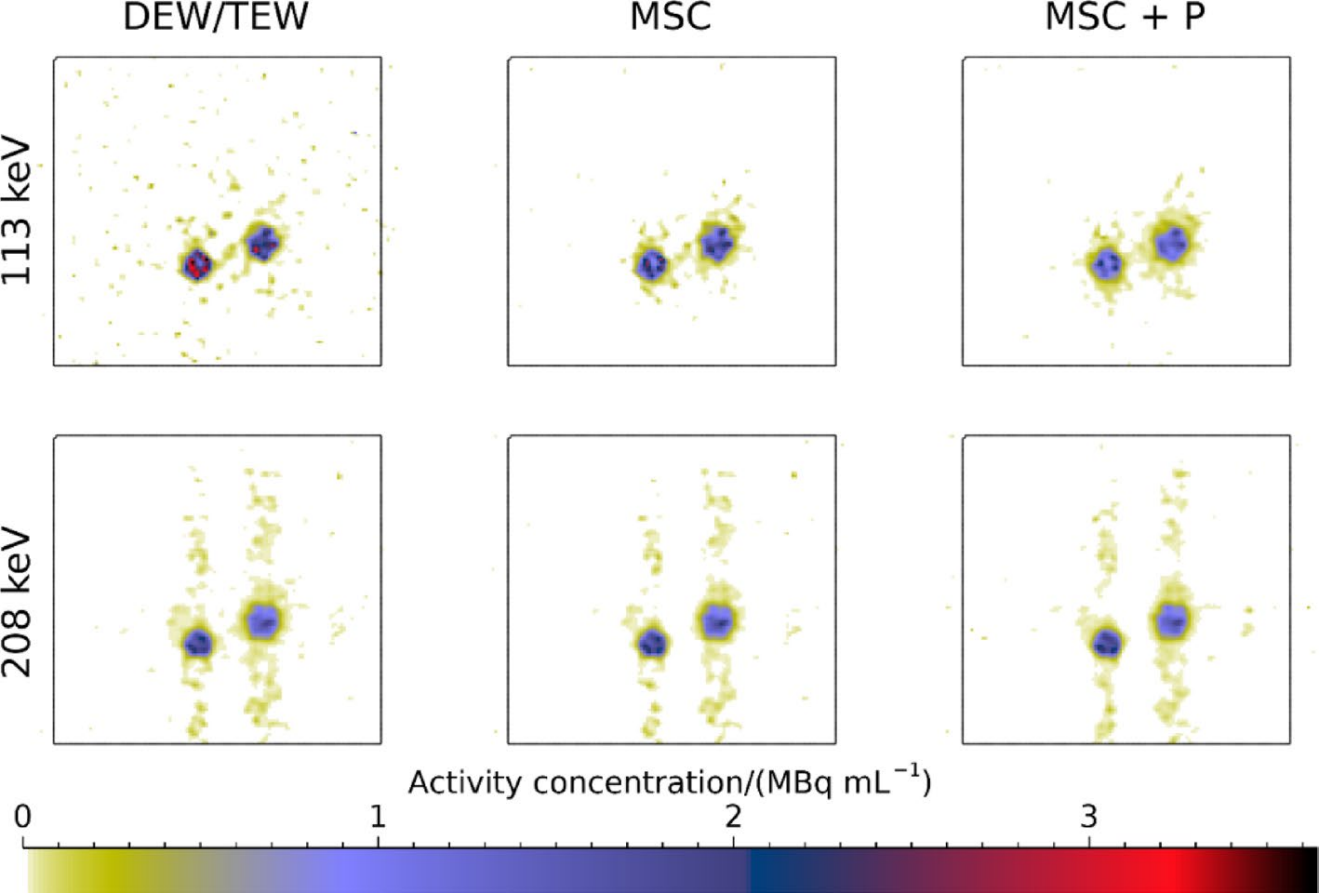
Sagittal slice for the anthropomorphic phantom with cold background reconstructed with 10 iterations (10 subsets). Top row shows images reconstructed with 113 keV (top left: DEW, middle: MSC, top right: MSC + P) peak and bottom row shows images reconstructed with 208 keV (top left: TEW, middle: MSC, top right: MSC + P). Images for DEW and TEW are for data from Stenvall et al. [2] and are included for comparison

Examples of reconstructed images of the anthropomorphic phantom with radioactive and non-radioactive liver-background are presented in Figs. 5 and 6. For 113 keV with warm background reconstructed with MSC and MSC + P, the liver background appears more uniform compared with TEW. The modified scatter compensation methods reduce residual signal outside the liver. There are clear streaks in the axial direction visible for images at 208 keV, particularly for the images with non-radioactive background.

**Fig. 7:**
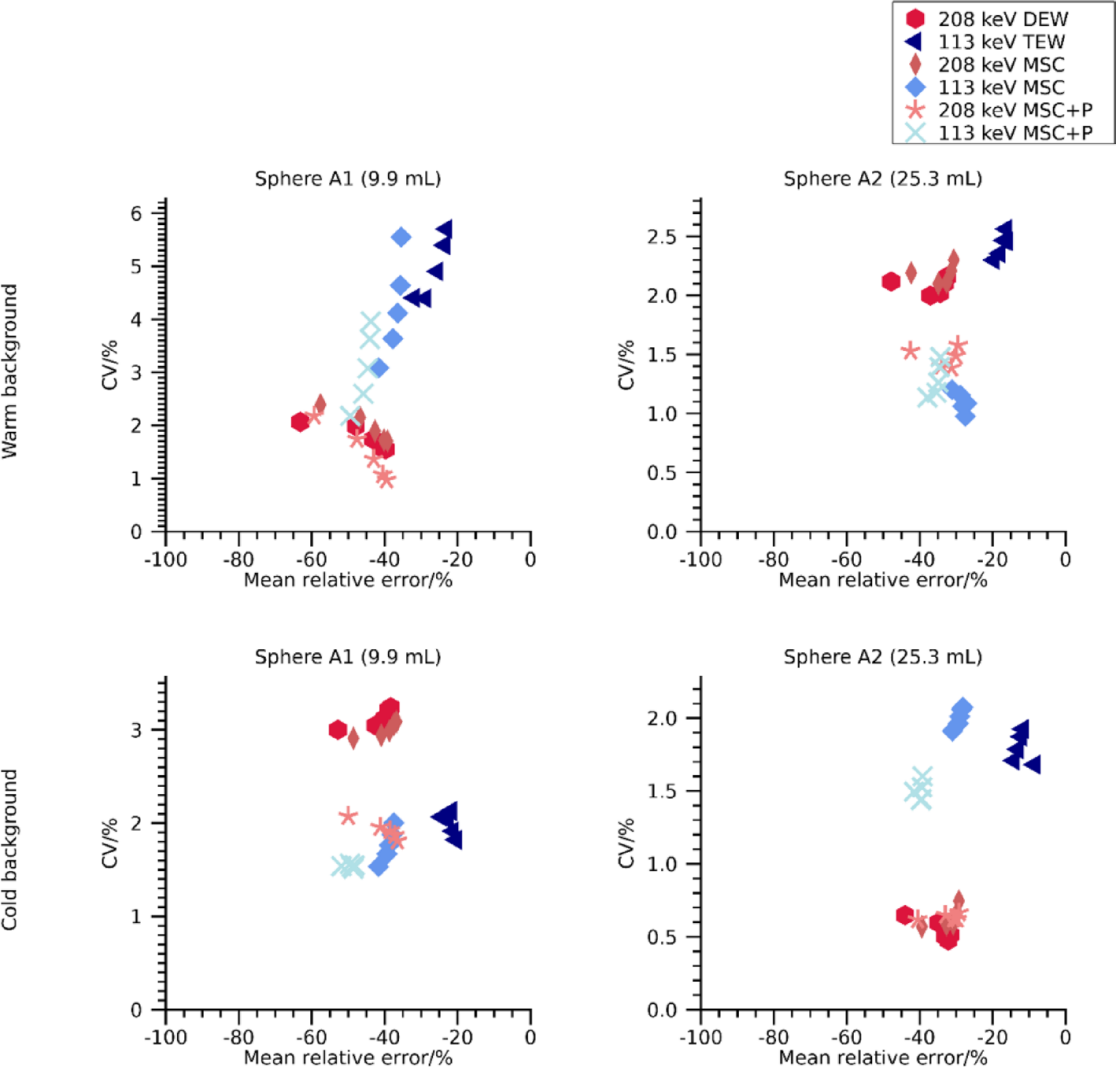
Coefficient of variation versus mean relative error for the anthropomorphic phantom where each symbol represents a specific number of iterations. Top row shows measurements performed with warm background and bottom row shows measurements performed with cold background. Note that the ordinate is individual for each subplot. Data for DEW and TEW are from Stenvall et al. [2] and are included for comparison

Mean relative error and CV for the two spheres are presented in Fig. 7 and numerical data in presented in Table 3. These findings align with the result reported for the NEMA phantom where consistent results for mean relative error and CV are obtained for DEW, MSC and MSC + P for the 208 keV peak for both warm and cold liver background. For the 113 keV peak, MSC and MSC + P are overall associated with slightly better CV. However, TEW yields the lowest mean relative error for warm background (mean relative error (CV): − 16.3% (2.6%) (TEW), − 27.1% (1.1%) (MSC), − 34.3% (1.5%) (MSC + P) for 30 iterations). For cold liver background, mean relative errors for the largest sphere with TEW results in a value of − 12%, whereas for the MSC the mean relative error is − 28% and MSC + P is − 39%, as stated in Table 3.

**Fig. 8:**
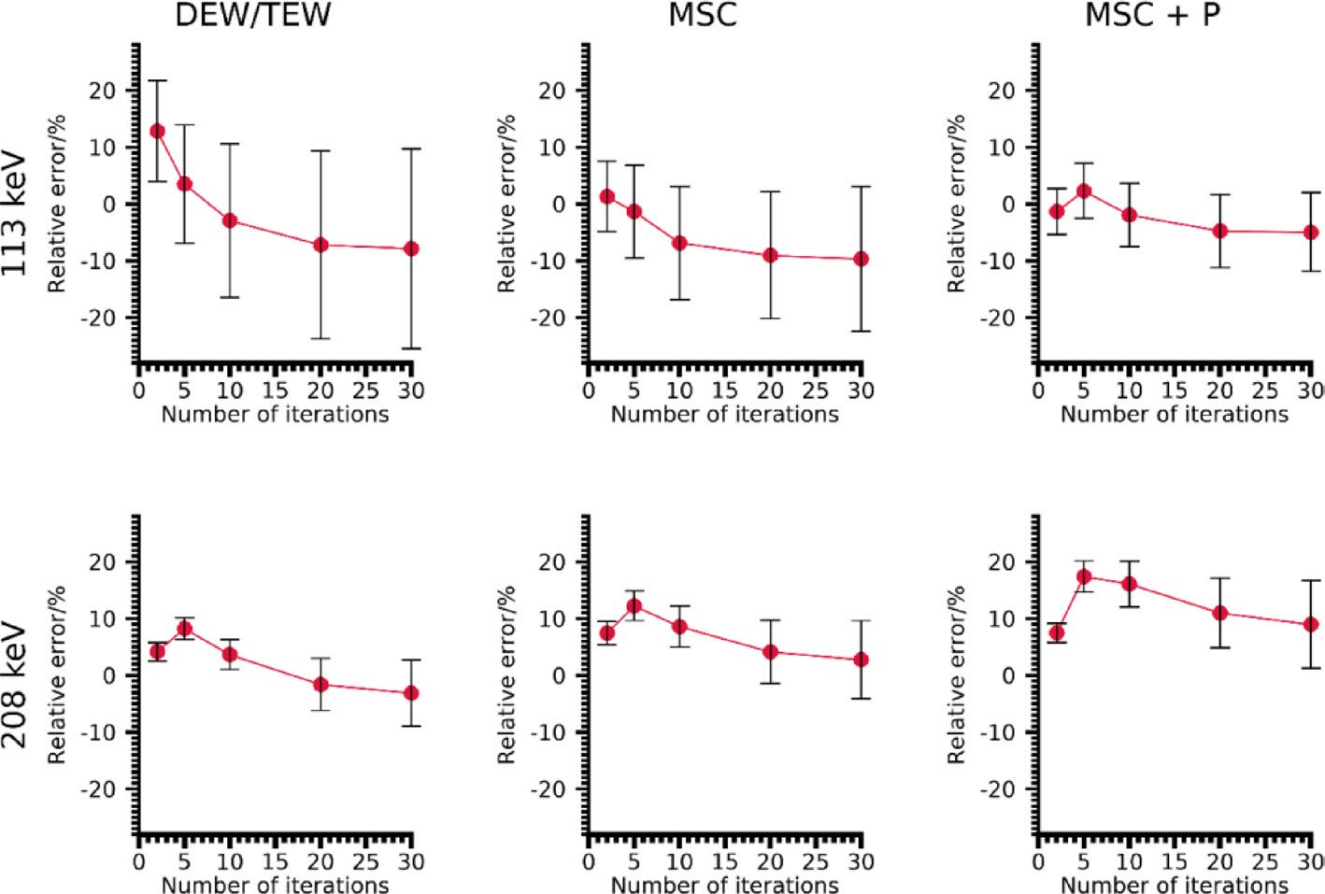
The relative error with standard deviations as a function of number of iterations for a 10 ml VOI in the liver compartment of the warm anthropomorphic phantom

Figure 8 shows mean relative error and SD as a function of number of iterations for a 10 ml spherical VOI placed in the liver background. For the 208 keV peak the mean relative error and CV for DEW at 30 iterations were − 3.1% and 6.0%. The modified scatter compensation methods (MSC and MSC + P), on the other hand, resulted in similar relative error, although slightly larger for MSC + P while the SD remained comparable over iterations. For the 113 keV peak TEW and MSC show similar mean relative errors, however TEW shows larger variabilities compared to MSC. Mean relative errors (CV) for DEW were − 7.9% (19.1%) and − 9.7% (14.1%) for MSC for 30 iterations. MSC + P perform better than all other scatter compensation methods with a mean relative error of about zero followed by an underestimation of about a few percentage points for increasing number of iterations. Mean relative error and CV for MSC + P were − 5% (7.3%) for 30 iterations.

**Fig. 9:**
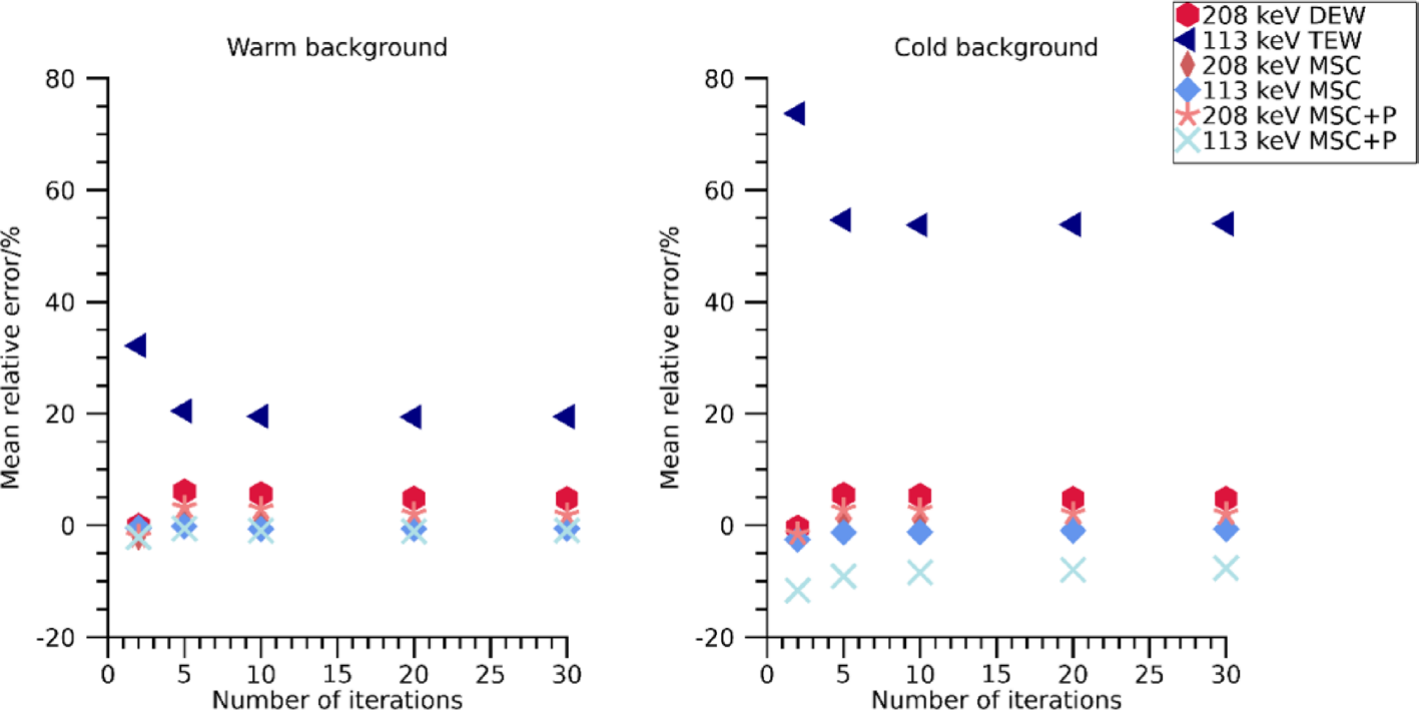
Mean relative error of total activity in the anthropomorphic phantom as a function of number of iterations. Data for DEW and TEW are from Stenvall et al. [2] and are included for comparison

Mean relative error in estimation of total activity in the anthropomorphic phantom as a function of number of iterations are shown in Fig. 9. As demonstrated for the NEMA phantom in Fig. 4, the total activity stabilizes after approximately five iterations (i.e., 50 updates). For the warm liver background TEW overestimates the activity concentration by an average 20%, whereas MSC and MSC + P have mean relative errors close to zero. For the 208 keV peak and all scatter compensation methods (DEW, MSC and MSC + P), the mean relative error ranges from 0% to 5%. For the cold liver background, higher mean relative errors are generally observed for all scatter compensation methods, where the 113 keV peak produces mean relative errors of + 55, − 10 and ~ 0 for TEW, MSC, MSC + P, respectively. For the 208 keV peak similar values as for the warm liver background are obtained with a mean relative error ranging from 0 to 5% for all scatter compensation methods used (DEW, MSC and MSC + P).

### Estimated total activity for extended acquisition time

**Fig. 10:**
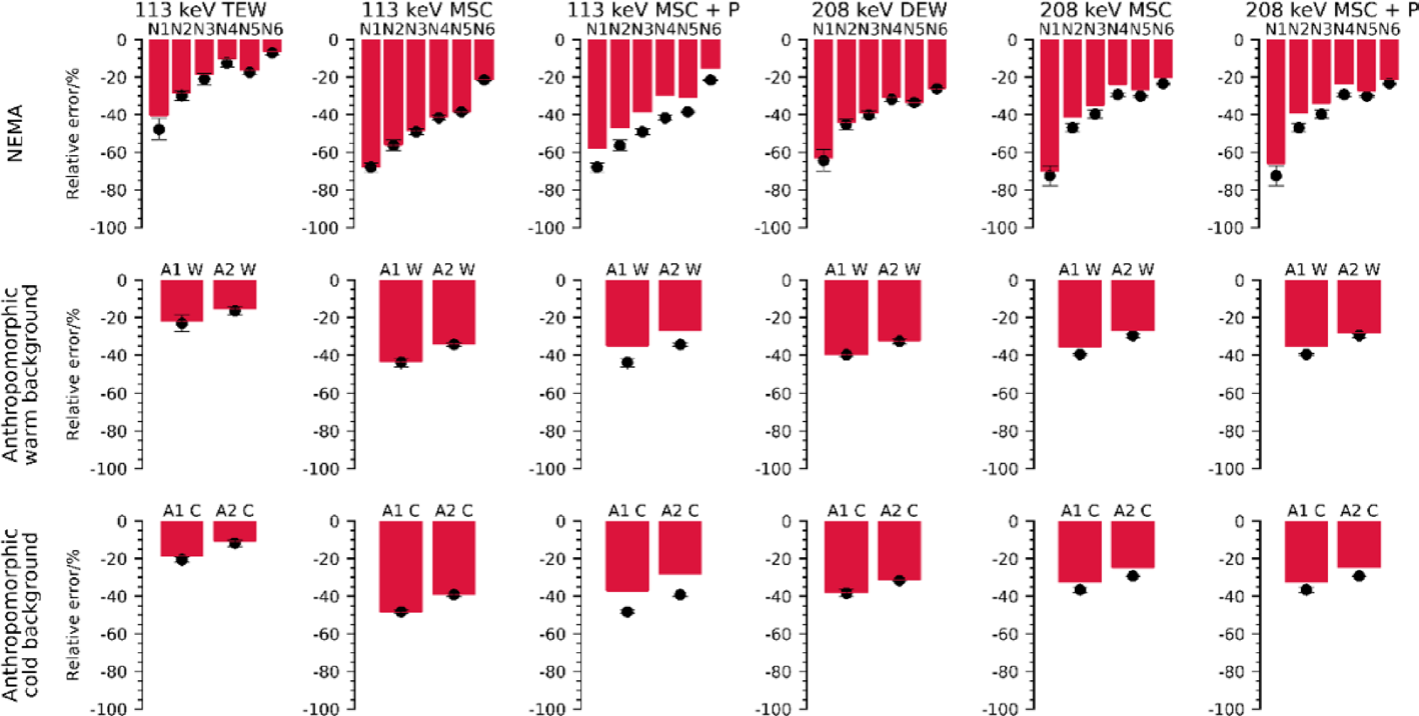
Mean relative error and standard deviations in NEMA spheres for total activity in 10-minutes time frames (black circles) and relative error for full-time data (bars) (top row). Corresponding estimations for the spheres in the anthropomorphic warm and cold liver background in the middle and lower row, respectively. Data for DEW and TEW are from Stenvall et al. [2] and are included for comparison

Figure 10 shows the mean relative error and standard deviation for each sphere reconstructed with 10 min time frame (black circles) compared to the relative error for reconstructed data using full-time data (bars) for the NEMA and anthropomorphic phantom with cold and warm background. For NEMA phantom for the 113 keV peak MSC, there is no noticeable difference in relative error when comparing short versus long acquisition time (mean relative error ranging from − 67% for N1 to − 25% for N6). However, for the MSC + P a larger difference is observed, where the relative error is reduced by 10% points for the long timeframe compared to the short timeframe (relative error ranging from − 67% (10 min time frames) to 57% (60 min time frame) for N1 and − 25% (10 min time frames) to 15% (60 min time frame) for N6). The same observation can be made for the anthropomorphic phantom with cold and warm liver background, where a decrease in mean relative error of approximately 10% points is obtained for MSC + P for the 113 keV peak (mean relative error: -35% (10 min time frames) and 25% (60 min time frames) for A2W and − 40% (10 min time frames) and − 30% (60 min time frame) for A2C. For the 208 keV images the difference is a few percentage points when comparing short versus long times for all scatter compensation methods for both the NEMA phantom and anthropomorphic phantom with cold and warm liver background.

## Discussion

In our previous study [2], we demonstrated lower mean relative errors for imaging at 113 keV with TEW compared with 208 keV with DEW (also shown in Figs. 3 and 7 in the current paper), but that this lower bias in concentration was accompanied by a gross over-estimation of total activity in the phantom (also shown in Figs. 4 and 9 in the current paper). The difference between estimates for 113 and 208 keV is smaller for the modified scatter compensation methods. In general, the modified methods (MSC and MSC + P) for 113 keV are associated with a higher bias but a slightly better precision for activity-concentration estimates than the TEW method. The most striking difference, however, is the substantially better estimation of total activity with the modified methods. In Stenvall et al. [2], we judged the better recovery of activity concentration for 113 keV as a genuinely better property rather than merely being an indirect result of overestimation of total activity. This was based on that we were able to reduce bias in total activity by increasing the total acquisition time while keeping the low bias for concentration estimates. The results in the current study to some extent contradict that conclusion, as the modified scatter-compensation methods, which are theoretically better underpinned than standard TEW for CZT-based systems and are quite successful in reducing the total-activity error, but also leads to worse activity-concentration estimates.

Estimates for the 208 keV energy windows are relatively unaffected by the introduction of MSC compared with the older DEW implementation. This is due to the fact that the old DEW method already accounted for the tail effect (a setting that could not be disabled in the reconstruction software), and thus the main difference between DEW and MSC for 208 keV is the use of different scatter-window widths, following updated recommendations from the system manufacturer. The similarity between estimates from these two methods suggests that the exact definition of the scatter window is not the main issue with respect to accuracy of activity-concentration estimates. There is little consistent difference in activity concentration estimates between 113 and 208 keV when employing the modified scatter methods, although the largest sphere in the NEMA phantom does reach a better recovery for the activity concentration estimates for 113 keV. A further problem for imaging at 208 keV is the issue of penetration at this energy [11], which is also visible in, e.g., Fig. 6, despite the employment of explicit penetration compensation in the reconstruction.

The rationale behind the MSC + P method is to utilize the primary signal in the scatter windows to boost the signal-to-noise ratio. However, as the acquired signal in these scatter windows have a poor scatter-to-primary ratio, the extra primary signal will be uncertain and thus risk increasing systematic errors. The trend observed for activity-concentration estimates in Figs. 3 and 7 for the NEMA and anthropomorphic phantom shows larger mean relative errors for MSC + P compared with MSC, although at the gain of a slightly lower CV for both peaks. These two observations could potentially explain the low-signal background seen centrally for the NEMA phantom when employing MSC + P at 113 keV in Fig. 1 indicating a misplacement of the signal. The combination with larger mean relative error (Figs. 3 and 7) and the misplaced signal observed in Fig. 1 for MSC + P appears to provide little reason to employ the MSC + P method for estimation of activity-concentration in high-concentration regions. Conversely, Fig. 8 indicates a potential advantage of MSC + P compared with MSC and TEW for estimation of the liver background activity concentration, where this method achieves substantially better repeatability over timeframes than other methods with no major difference in systematic error for 113 keV. These results are also in line with the images presented in Fig. 5, where the MSC + P images appear less noisy than other methods for 113 keV. Thus, when applied to the liver background, MSC + P may play a role in improving the estimation of lower activity concentrations, owing to its capacity to reduce random errors. The advantages are apparent only for the 113 keV peak, in contrast, no pronounced differences are observed for the 208 keV peak when using MSC + P.

Much research around imaging of ^177^Lu on ring-configured CZT gamma-cameras has focused on the ability to shorten acquisition times [12]. In Stenvall et al. [2], we argued that such reduction should be carried out cautiously and that similar opportunities to reduce acquisition times are already available on standard gamma-cameras based on Anger logic. The better preservation of total activity for 10-minute acquisitions for the modified scatter-compensation methods, to some extent, relieves the problems indicated for short acquisition times. However, the MSC + P method demonstrates better activity-concentration estimates when the full acquisition time of 1 h is used compared with the shorter ones. Thus, there still appears to be a risk of introducing bias if the acquisition time is aggressively reduced.

An issue related to the reduction of acquisition time is the definition of optimality for quantitative SPECT, which is fundamentally different from diagnostic SPECT [13]. In Gustafsson et al. [14], we adopted a Pareto-optimization approach for ^177^Lu activity quantification due to the inherent conflict between bias and precision for SPECT reconstruction, and thus optimality cannot be uniquely defined without imposing further assumptions. The presentation of results, with bias and precision presented in Figs. 3 and 7 is motivated by the same issues, where we do not believe that these two aspects can be considered separately and superiority, in principle, can only be claimed when lower bias is obtained for the same precision or vice versa. Another aspect is the need for a relatively large number of iterations in order to counter-act the non-linearity of the OS-EM algorithm [15]. In view of these aspects, and from the results presented in the current paper, we argue for a relatively large number of updates to be used in tasks involving the estimation of activity concentration in small volumes such as tumours. The question of the distribution between number of iterations and number of subsets for reconstruction with the StarGuide system has been studied in some detail by Danieli et al. [11] and does not appear to have a major effect on quantitative performance. Thus, we believe that results derived in the current study for 10 subsets are generalizable also to other reasonable choices of subset-size.

One major limitation of the current study is its reliance on the camera manufacturer’s software for reconstruction and hence also for implementation of scatter-compensation methods. Thus, we do not have full insight into the implementation details of the modified scatter-compensation methods, which also limits the scope of the conclusions that can be drawn from experiments. This is in line with, what we believe, is a general problem for the literature on SPECT for ring-configured CZT system, where it has proven difficult to separate the effects of detector material, camera geometry, and software. For the moment, we can only make statements that are specific to the StarGuide system (including software), and the generalizability to other systems is uncertain and requires further research.

Another aspect to consider for the future is the study of model-based scatter compensation for ring-configured SPECT systems. Window-based methods have indeed been proven useful for estimating scatter, but tend to deteriorate the signal-to-noise ratio and suffer from an inherent conflict between noise and bias with respect to window width [16]. In light of the extra complexity of modified scatter-compensation methods appropriate for pixelized CZT detectors compared with the original DEW and TEW methods, taking the step to a model-based approach could be proven advantageous, at least for quantitative tasks.

## Conclusions

Modified scatter compensation methods that account for the low-energy primary tail improve the preservation of total activity for quantitative ^177^Lu SPECT using ring-configured CZT SPECT systems compared with original window-based scatter compensation for both 113 and 208 keV. The improvement in activity-concentration accuracy is modest, and even slightly worse for 113 keV in small-volume high activity-concentration regions. Using the estimated primary signal in scatter windows to increase the signal-to-noise ratio is not advisable for the estimation of high activity concentrations but may have a role for the estimation of low activity-concentrations, especially for 113 keV.

## Data Availability

Data are available upon reasonable request to the corresponding author.

## Abbreviations

CV: Coefficient of variation
CZT: Cadmium zinc telluride
DEW: Dual energy window
HW: High window
LW: Low window
MRE: Mean relative error
MSC: Modified scatter correction
MSC + P: Modified scatter correction + primaries
OSEM: Ordered subset estimation maximization
PW: Primary window
SD: Standard deviation
SPECT: Single photon emission computed tomography
TEW: Triple energy window
VOI: Volume of interest
